# Factors Influencing the Sample Adequacy of Ultrasound-Guided Fine-Needle Aspiration from Solid Thyroid Nodules for Liquid-Based Cytology: A Demographic, Sonographic, and Technical Perspective

**DOI:** 10.3390/medicina58111639

**Published:** 2022-11-13

**Authors:** Ying Fu, Yan Sun, Qianqian Pei, Xiaobo Han, Wen Qin, Fang Mei, Shi Tan, Ligang Cui

**Affiliations:** 1 Department of Ultrasound, Peking University Third Hospital, Beijing 100191, China; 2 Department of Ultrasound, Tongxiang First People’s Hospital, Tongxiang 314500, China; 3 Department of Ultrasound, Qinhuangdao Third Hospital, Qinhuangdao 066000, China; 4 Department of Ultrasound, Xiangyang Central Hospital, Affiliated Hospital of Hubei University of Arts and Science, Xiangyang 441021, China; 5 Department of Pathology, Peking University Third Hospital, School of Basic Medical Sciences, Peking University Health Science Center, Beijing 100191, China

**Keywords:** factor, fine-needle aspiration, liquid-based cytology, sample adequacy, thyroid nodule

## Abstract

*Background and Objectives*: To identify factors that influence the sample adequacy of solid thyroid nodules based on ultrasound-guided fine-needle aspiration (FNA) with subsequent liquid-based cytology. *Materials and Methods*: We retrospectively reviewed 855 patients who underwent ultrasound-guided FNA at our hospital between July 2019 and July 2020. The final analysis included 801 solid thyroid nodules in 801 patients. After reviewing the demographic data, ultrasonic features, and FNA technique-related factors, we defined 14 potential variables. For cytological results, the Bethesda categories II–VI were defined as adequate sample results. Univariate and multivariate analyses were performed to identify factors that influenced sample adequacy. *Results*: The adequate sample rate was 87.1%. The univariate analysis showed that four factors were related to adequate sampling in patients with thyroid FNA. These factors included age (*p* < 0.001), nodule orientation (*p* = 0.0232), calcification (*p* = 0.0034), and operator experience (*p* = 0.0286). After the multivariate analysis, five independent factors were identified to improve the diagnostic results of FNA for solid thyroid nodules: (1) the presence of Hashimoto’s thyroiditis (odds ratio (OR) = 1.810; 95% confidence interval (CI): 1.076–3.045; *p* = 0.0254), (2) a taller-than-wide orientation (OR = 2.038; 95% CI: 1.260–3.296; *p* = 0.0037), (3) the presence of calcification (OR = 1.767; 95% CI: 1.115–2.799; *p* = 0.0153), (4) four needle passes to obtain material (OR = 1.750; 95% CI: 1.094–2.799; *p* = 0.0196), and (5) an experienced operator (OR = 0.561; 95% CI: 0.319–0.987; *p* = 0.0451). *Conclusions*: A taller-than-wide orientation, the presence of calcification, and the presence of Hashimoto’s thyroiditis were found to affect the sample adequacy of ultrasound-guided FNA with liquid-based cytology. The sample adequacy could be improved when FNA is performed with four needle passes by experienced doctors.

## 1. Introduction

Thyroid nodules are frequent pathologies of the endocrine system with an increasing prevalence (range, 19–70%) in ultrasound examinations. Of these, malignancies account for 5–15% [1,2,3]. In China, the age-standardized incidence of thyroid cancer was 3.21/105 in 2005 and increased to 9.61/105 in 2015 [1]. Given this high incidence, the malignant nodules should be distinguished from the benign nodules. In the clinical guidelines of various countries regarding the diagnosis and treatment of thyroid nodules and differentiated thyroid cancers, fine-needle aspiration (FNA) is recommended as the method with the highest sensitivity and specificity for the preoperative evaluation of benign and malignant thyroid nodules [2,3,4,5]. Globally, the preoperative evaluation and clinical treatment of thyroid nodules are mainly guided by FNA. FNA prevents unnecessary surgery on benign lesions and guides the treatment of malignant lesions. Therefore, obtaining accurate FNA results is important. The FNA sensitivity and specificity are 76–98% and 71–100%, respectively. For experienced doctors, the accuracy can reach 90% but remains below 100% [6].

Moreover, FNA has several limitations. The primary factor affecting its clinical application is the high rate of nondiagnostic (ND) samples [7,8]. Several studies investigated the factors associated with ND rates, most of which focused on the rapid on-site cytological evaluation and cystic components of the nodule [6,7,9,10]. However, many hospitals cannot perform on-site cytological diagnoses. Furthermore, compared to the conventional smear (CS) technique, liquid-based cytology (LBC) prevents interference from blood cells and is simple and easy to perform. Cystic or spongiform nodules are generally considered to be benign [5]. Approximately 5% of partially cystic nodules are malignant [11]. The probability of malignancy is significantly higher in solid nodules than in cystic nodules [5,11]. Thus, we aimed to assess the factors that influence the FNA sample adequacy rates of solid thyroid nodules in LBCs.

## 2. Materials and Methods

### 2.1. Ethics Approval

This study was approved by the Peking University Third Hospital Medical Science Research Ethics Committee (Ethics Approval No. 2022-IRB-422-01, date of approval April 2022). Given the retrospective nature of this study and the anonymized data analysis, the requirement for informed consent was waived.

### 2.2. Participants

**Figure 1:**
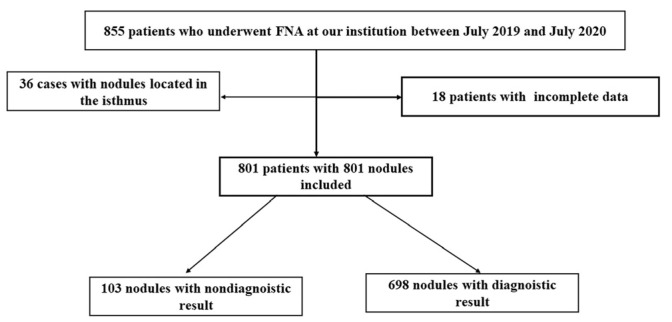
Flow diagram of the selection process and analysis of participants in this study. FNA, fine-needle aspiration.

We retrospectively analyzed patients who underwent FNA at our institution between July 2019 and July 2020. The included patients met the following criteria: (1) initial ultrasound-guided FNA, (2) no history of thyroid ablation before FNA, (3) FNA specimen evaluation and adequate medical records available, and (4) targeted nodules located in bilateral lobes. Exclusion criteria were (1) cystic or mixed cystic nodules and (2) nodules located in the isthmus. The final analysis included 801 thyroid nodules from 801 patients (Figure 1).

### 2.3. Clinical Data Review

Data on patients’ clinical information (sex and age), laboratory results (serum thyroid peroxidase antibody (TPOAb) and thyroglobulin antibody (TgAb) levels), ultrasound examination results, and histology results were reviewed if available in electronic medical records. Hashimoto’s thyroiditis (HT) was histopathologically confirmed in patients who underwent surgery or based on elevated serum TPOAb/TgAb levels combined with sonographic identification of diffusely heterogeneous parenchyma in those who did not have surgery [12].

### 2.4. Ultrasonic Evaluation

Sonographic examinations and ultrasound-guided FNA were performed using three commercial ultrasound devices (Logic E9, GE Healthcare, Chicago, IL, USA; Siemens ACUSON S3000, Siemens Healthineers, Erlangen, Germany; Samsung RS80A, Samsung Medison Co., Ltd., Seoul, Korea) with high-frequency linear array probes. Usually, a 3–12 mHz high-frequency linear-array probe was selected for superficial lesions. A 2–9 mHz linear-array probe or 3.0–5.0 mHz convex-array probe can also be used for better visualization of deeper lesions. Maximum nodule size, nodule distribution, nodule location, nodule orientation, margin, echogenicity, calcification type, and vascularity were recorded.

**Figure 2:**
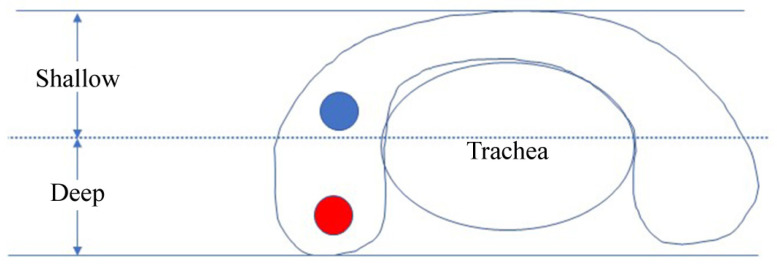
Sketch of shallow and deep nodules. A line is drawn at the midpoint of the distance between the highest and lowest points of the thyroid gland on a cross-section. Any nodule above this line is defined as a shallow nodule, and all others are defined as deep nodules.

Nodule distribution describes whether the tumor was in the left or right lobe, whereas tumor location refers to whether the tumor was in the upper or lower part of the lateral lobe on cross-sections. Nodules above or below the midpoint line between the lowest and highest points of the thyroid gland on cross-sections were defined as shallow or deep nodules, respectively (Figure 2). Microcalcifications were defined as bright stippled hyperechogenic dots of ≤1 mm size with/without shadow. Macrocalcification within a nodule was defined as any calcification not categorized as microcalcification. Peripheral calcification, which might appear as a continuous or discontinuous ring or arc, was defined as a calcification located at the nodule periphery involving more than one-third of the margin [5]. Mixed calcification was defined as the presence of both microcalcification and macrocalcification, regardless of peripheral calcifications. 

Lesions without intranodular flow signals on color Doppler ultrasound were considered avascular. Tumor vascularity was classified as minimal, moderate, or marked [13]. One to two pixels containing flow (usually <1 mm in diameter) were considered minimal. If a major vessel or several small vessels were visualized, blood flow was categorized as moderate. Tumors with more than four intranodular vessels on color or power Doppler imaging were classified as marked.

### 2.5. Ultrasound-Guided FNA

**Figure 3:**
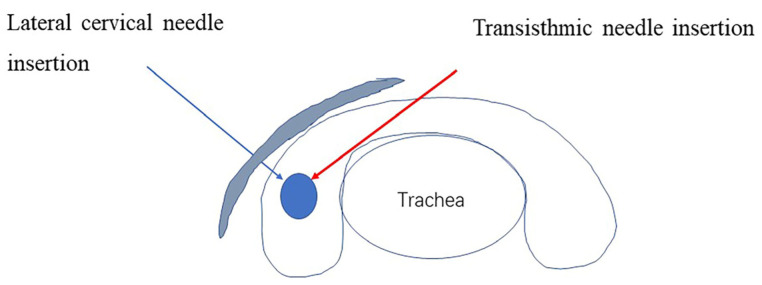
Sketch illustrating the needling direction.

The FNA procedure was established according to expert consensus and Chinese guidelines [14]. FNAs were performed by interventional ultrasound physicians. A 25 G needle (Hakko, Chikuma, Japan) was used under ultrasound guidance, and three or four passes were performed for each nodule according to the guidelines. Each pass included 6–7 to-and-from needle movements over 5–10 s per nodule. Using a freehand parallel approach, aspirations were conducted at different angles and within different nodule regions to obtain representative samples. Needle insertion was directed either transisthmic or lateral cervical (Figure 3).

Samples were primarily obtained without suction via the capillary method. The aspirated material was submerged in a vial containing methanol-based preservatives (Cytoprep solution, Cheng Zhi Guang Hui, Beijing, China) and sent to the cytopathology laboratory for centrifugation and staining (SurePath, TriPath Imaging, Burlington, NC, USA). One slide per lesion was used, and each slide had a thin layer of cells concentrated in the central slide area. 

### 2.6. Cytopathology

An experienced pathologist reported the cytopathological findings according to the Bethesda criteria: (I) nondiagnostic (ND) (fewer than six groups of well-visualized follicular cells, each group containing less than 10 well-preserved epithelial cells); (II) benign lesion; (III) atypia of undetermined significance/follicular lesion of undetermined significance (AUS/FLUS); (IV) follicular neoplasm/suspicious for follicular neoplasm (FN/SFN); (V) suspicious for malignancy; and (VI) malignancy [15]. In this study, Bethesda II–VI were determined to indicate adequate sample results. 

### 2.7. Statistical Analysis

Continuous data are expressed as the mean ± standard deviation, categorical data as counts (percentages). Fourteen potential prognostic factors were considered in this study, and their classification cores are listed in Table 1.

Patient-related factors included sex, age, and presence of HT. Tumor-related factors included maximum tumor size, nodule distribution, nodule location, tumor orientation, margin, echogenicity, calcification, and vascularity. Technique-related factors included needling direction, number of needle passes, and operator experience. Operator experience was divided into experienced (>60 FNAs/year, >1 year) and inexperienced (<60 FNAs/year, <1 year).

The *t*-test was used to analyze continuous variables, and the chi-square test was used to analyze categorical variables. The U test was used if data did not conform to a normal distribution. Univariate and multivariate logistic regression analyses were performed. All statistical analyses were performed using R software, with *p* < 0.05 indicating statistical significance.

## 3. Results

### 3.1. Adequate Samples

We evaluated 801 nodules from 801 patients comprising 235 men and 566 women, with an average age of 42.7 ± 13.7 years. The average lesion size was 12.3 ± 6.5 mm. Of 565 nodules with calcifications, 259 had microcalcifications, 149 macrocalcifications, 14 peripheral calcifications, and 145 mixed calcifications. A total of 698 cases had Bethesda II–VI results, whereas 103 cases had ND findings, resulting in 87.1% adequate samples and a 12.9% ND rate (Table 2).

### 3.2. Univariate Analysis

Univariate analysis showed that four factors were related to the adequate sample rate in thyroid FNAs. These factors included age (*p* < 0.001), nodule orientation (*p* = 0.0232), calcification (*p* = 0.0034), and operator experience (*p* = 0.0286). Patient sex, presence of HT, maximum nodule size, nodule distribution, nodule location, margin, echogenicity, and vascularity were not related to the adequate sample rate (*p* > 0.05, Table 3).

### 3.3. Multivariate Analysis

Because some factors may interact, odds ratios (ORs) were estimated for the diagnostic yield using multivariate logistic analysis. The results showed that HT was significantly associated with diagnostic cytology, nodule orientation, calcification, number of needle passes, and operator experience.

Regarding thyroid gland background, the presence of HT (OR = 1.810; 95% confidence interval (CI): 1.076–3.045; *p* = 0.0254) increased the probability of obtaining diagnostic results. For the nodule itself, a taller-than-wide shape (OR = 2.038; 95% CI: 1.260–3.296; *p* = 0.0037) and the presence of calcification (OR = 1.767; 95% CI: 1.115–2.799; *p* = 0.0153) increased the probability of adequate sampling. For technical factors, four needle passes (OR = 1.750; 95% CI: 1.094–2.799; *p* = 0.0196) were approximately 1.75 times more likely than three passes to achieve a conclusive diagnosis. Operator experience (OR = 0.561; 95% CI: 0.319–0.987; *p* = 0.0451) was an independent factor for the improvement of FNA-based diagnosis of solid thyroid nodules.

There were no significant differences in adequate sample rates based on sex, age, nodule echogenicity, nodule distribution, nodule position, nodule size, margin, or vascularity (Table 4). Regarding technical factors, adequate sample rates did not significantly differ between needling directions (*p* = 0.174).

## 4. Discussion

Currently, the cytological results dominate the diagnostic process in patients with thyroid nodules [16]. The benign FNA results help prevent unnecessary surgery and reduce it by 25% in patients with benign thyroid disease [17,18,19]. Although the prognosis of thyroid cancer is good, the cytological confirmation of malignancy facilitates choosing hemithyroidectomy versus total thyroidectomy and the extent of the lymph node dissection [7,20]. However, ND results have been reported in up to 33.6% of FNA cases, even under ultrasound guidance, with an overall value of 12.9% considering all studies in a meta-analysis [7,21]. The ND FNA rate of 12.1% in our study was similar to the average reported in the literature [4,6,7].

The usual management is to repeat FNA under ultrasound guidance. In a large-scale population meta-analysis, 16.8% of the ND patients with subsequent surgery had malignant nodules [7]. This malignancy rate is higher than that postulated in the guidelines [2,3,4]. Therefore, understanding the factors affecting FNA success rates is important for the diagnosis and treatment of thyroid nodules. Integrating the information of US-based risk stratification systems—for example, Chinese thyroid imaging reports and data systems (C-TIRADS)—may serve as a means to reduce the number of ND samples for appropriate nodule selection and pathological image interpretation [5]. The 2017 Bethesda System for Reporting Thyroid Cytopathology further emphasized that the pathological diagnosis of ND samples should be combined with ultrasonic data [15].

To improve the diagnostic yield of FNAs, many studies have examined the factors that contribute to the ND rate. HT, a common condition in this population, is an autoimmune disease characterized by lymphocytic gland infiltration and TPOAb and TgAb production, resulting in tissue destruction and progressive loss of thyroid function [22]. HT results in thyroid gland hardening, which lowers the efficiency of elastography in the diagnosis of benign and malignant nodules [23]. To date, only a few reports have discussed the relationship between the HT and FNA results. Hu et al. concluded that the presence of concurrent HT, whether clinically implied with antithyroid autoantibody positivity or pathologically confirmed, is unlikely to predispose an FNA diagnosis of thyroid nodules to ND or indeterminate [24].

Other studies have drawn contrasting conclusions [25,26], indicating that concurrent HT increases the likelihood of indeterminate cytological results (Bethesda grades I, III, and IV) in ultrasound-guided FNAs. However, their data suggest that the ND rate is decreased in the HT group, without reaching statistical significance. In a study of 9851 consecutive patients over 22 years, the ND rate in the HT group was 4.3% compared to 7.2% in the non-HT group. Although the lack of multivariate analysis is a limitation, the focus of this study was to determine whether the malignancy risk of nodules was significantly elevated in patients with HT [12].

In our study, the univariate analysis did not reveal a relationship between HT and the adequacy of FNA. According to multivariate analysis, the likelihood of diagnostic cytological results was significantly increased in patients with HT. We suspect that the effect of this factor is masked in the univariate analysis by the influence of other confounding factors such as patient age (*p* < 0.001 in the univariate analysis; Table 3). HT mostly occurs in middle-aged women, making age a potential confounder. The ND rate was higher in the >55-year-old group than in the ≤55-year-old group. Pathological HT lesions involve both thyrocytes and the interstitium around thyroid follicles. Lymphocytes come into close contact with thyrocytes and may mediate thyrocyte destruction. Moreover, the occurrence of large follicular cells and oxyphilic or Askanazy cells is frequently associated with HT [12,22,23]. We speculate that lymphocyte infiltration decreases the adhesion between follicular cells, resulting in increased capillarity. This may be the reason for the improved acquisition of optimal cellular material. Our study may be the first to investigate the real influence of the HT background on ND results and analyze possible reasons. However, this requires further verification using pathological and clinical data.

The effect of nodule orientation on cell volume is well-understood. After the needle tip had reached the target, specimens were collected using 6–7 movements to induce capillary formation along the long axis. A taller-than-wide shape increased the lifting length of the needle in the nodule. For deep nodules, the visualization of the needle along the long nodule axis improves the diagnostic rate compared to visualization along the short axis [27].

Whether calcifications affect ND rates remains controversial. Regarding the FNAs of calcified thyroid nodules, the ND rates range from 6.4% to 17.2%, and the rates for nodules with large calcifications are as high as 24.1% [28,29,30]. Annular calcifications are usually dense, large, and thick and surround or fill nodules. Thus, the biopsy needle may not enter the nodule core, resulting in insufficient aspirated parenchyma. However, various studies arrived at the opposite conclusion that FNA of thyroid nodules with macrocalcifications has a high diagnostic yield and accuracy [31,32]. In our study, the diagnostic cytology was significantly increased in nodules with calcifications. Our FNA technique facilitates obtaining as much material as possible from multiple angles. Therefore, microcalcifications and coarse calcifications have little effect on sample extraction. Further studies are required to clarify this.

Currently, FNAs are predominantly performed by experienced doctors, which can reduce ND incidence [6]. Doctors experienced in core needle biopsies may not be fully competent in cytological punctures because cytology uses different methods. Our research answered this question as operator experience independently improved the adequacy rate of FNAs in solid thyroid nodules (Table 4). Therefore, even experienced interventional sonographers who have not previously performed FNAs still need to be trained in FNA maneuvers and reviewed for at least 60 cases to ensure sufficient learning effects. With the massive growth of the FNA numbers in clinical practice, it is impossible for experienced doctors to complete all FNAs. Resident doctors should be performing FNAs primarily. The ND rates of thyroid FNAs progressively decrease with training, suggesting that early and continued participation in procedures throughout residency improves the outcomes [24]. Our results suggest that operator training and experience significantly affect the cellular sample adequacy. Simulation training among doctors who intend to perform FNAs may help increase the number of FNA cells.

The puncture technique is important for obtaining satisfactory specimens. Despite expert guidelines and consensus statements for FNAs, the number of punctures is not standardized among guidelines [2,3,4,5,6,7,14,33]. A consensus statement published by the Korean Society of Thyroid Radiology recommends two to three needle passes for each thyroid nodule. This number depends on the doctor’s experience and the ultrasound characteristics of the nodule [34]. A Chinese expert consensus and operation guideline recommends at least three punctures if on-site cytology evaluations are impossible [14]. The number of needle passes in a multicenter study ranged from 1 to 11, while the mean number necessary for adequate specimen acquisition was 3.8 ± 0.07 [34]. Some researchers have suggested that, with the increasing number of aspirations, the false-negative rate decreases [35]. However, the increased number of needle passes causes more complications and tissue injuries, e.g., papillary endothelial hyperplasia, hemorrhage, capsular distortion, fibrosis, cystic change, vascular thrombosis, vascular proliferation, and nodule infarction [36]. Furthermore, with the increasing number of attempts, discomfort and pain may increase in patients. Therefore, it is necessary to determine the optimal number of needle passes to obtain satisfactory cellular material. We selected three and four needle passes based on various guidelines and studies. The results showed that, compared to three punctures, four punctures significantly improved satisfactory cell sampling by approximately 1.75 times. Thus, four needle passes is a better choice for solid thyroid nodules if an on-site cytological diagnosis is impossible.

Moreover, we found that nodule echogenicity, nodule distribution, nodule position, nodule size, margin, and vascularity did not affect the cytology results. Increased vascularity is correlated with false-negative FNA and ND results in CS because of blood contamination [32]. An on-site adequacy assessment is generally associated with improved CS adequacy [10,32]. However, this describes ideal conditions. An on-site cytopathologist may not always be available for the immediate evaluation of aspirates, as in our hospital. Furthermore, if CS is performed with an on-site evaluation, the number of aspirations increases considerably, and the complication rate may also increase. This problem is addressed by using the LBC and thin-layer techniques. LBC slides require no manual smearing skills as the process is automated. This technique provides a cleaner background with fewer air-drying artifacts, and the ND rates do not differ between CS and LBC [37]. Therefore, the increased blood flow did not reduce the success rate of cell sampling in our study [38]. LBC allows the application of new techniques such as immunocytochemistry and gene detection to the same sample, facilitating further evaluation [39].

This study has several limitations. First, it is a retrospective study, and the data should be confirmed through prospective interventional studies. Second, only 25 G needles were used. According to the guidelines [6,14], 21 to 27 G needles can be used for FNA. The effects of different needle sizes on cell sampling were not considered. Third, to study the effects of needle entry directions on cell sampling, isthmic nodules were excluded. Satisfactory cytological results were obtained in 36 cases of isthmic nodules, which may be related to their shallow location and deep tracheal support, warranting further studies. Finally, in the current study, solid thyroid nodules that were taller than wide or had calcifications were associated with higher rates of cytological adequacy. However, these features are also risk factors for malignancy. The definition of adequate cytology in the current study is “Bethesda categories II–VI.” The cytology result is not compared with surgical pathology or core needle biopsy results. If malignancy pathology results are eventually obtained, the cytology category of Bethesda II (benign) would be considered “adequate” in the current study but still not acceptable in a clinical situation.

## 5. Conclusions

In conclusion, solid thyroid nodules that were taller than wide, had calcifications, or had concurrent HT were associated with higher rates of cytological adequacy in LBC after ultrasound-guided FNA. Furthermore, experienced operators and four needle passes were significantly associated with cytological cellularity.

## Figures and Tables

**Table 1:** Potential variables and their class scores in patients that underwent ultrasound-guided fine-needle aspiration for solid thyroid nodules.

Variables	Definition	Class Scores
X1	Sex	Male (0), female (1)
X2	Age (in years)	≤55 (0), >55 (1)
X3	Hashimoto’s thyroiditis	Absence (0), presence (1)
X4	Tumor size (in mm)	≤10 (0), >10 (1)
X5	Nodule distribution	Right lobe (0), left lobe (1)
X6	Nodule position	Shallow (0), deep (1)
X7	Echogenicity	Hypoechogenicity (0), isoechogenicity, or hyperechogenicity (1)
X8	Orientation	Parallel (0), taller-than-wide shape (1)
X9	Margin	Smooth (0), ill-defined (1)
X10	Calcification	None (0), microcalcification, or macrocalcification (1)
X11	Vascularity	Avascular or with minimal blood flow (0), moderate or marked intranodular vascularity (1)
X12	Number of needle passes	3 times (0), 4 times (1)
X13	Experience of the operator	Inexperienced (0), experienced (1)
X14	Needling direction	Lateral cervical needle insertion (0), transisthmic needle insertion (1)

**Table 2:** Fine-needle aspiration cytology of 801 patients with 801 nodules.

Cytology	Case Number
ND	103
Benign	33
AUS/FLUS	159
FN/SFN	33
SM	70
Malignant	403

ND, nondiagnostic; AUS/FLUS, atypia of undetermined significance/follicular lesion of undetermined significance; FN/SFN, follicular neoplasm/suspicious for follicular neoplasm; SM, suspicious for malignancy.

**Table 3:** Univariate analysis of risk factors for the diagnostic results in ultrasound-guided fine-needle aspiration of solid thyroid nodules.

	ND (*n* = 103)	Diagnostic Result (*n* = 698)	*p* Values
Sex			
Male	28 (27.2%)	207 (29.7%)	0.6070
Female	75 (72.8%)	491 (70.3%)	
Age (years)			
≤55	53 (51.5%)	517 (74.1%)	<0.001
>55	50 (48.5%)	181 (25.9%)	
Hashimoto’s thyroiditis			
Without	27 (26.2%)	154 (22.1%)	0.3470
Present	76 (73.8%)	544 (77.9%)	
Echogenicity			
Hypoechogenicity	93 (90.3%)	660 (94.6%)	0.0887
Isoechogenicity or hyperechogenicity	10 (9.7%)	38 (5.4%)	
Nodule distribution			
Right	53 (51.5%)	397 (56.9%)	0.3010
Left	50 (48.5%)	301 (43.1%)	
Nodule position			
Shallow	65 (63.1%)	463 (66.3%)	0.5190
Deep	38 (36.9%)	235 (33.7%)	
Nodule size (mm)			
≤10	55	348	0.5020
>10	48	350	
Orientation			
Parallel	65 (63.1%)	357 (51.1%)	0.0232
Taller-than-wide shape	38 (36.9%)	341 (48.9%)	
Calcification			
None	43 (41.7%)	193 (27.7%)	0.0034
Microcalcification or macrocalcification	60 (58.3%)	505 (72.3%)	
Shape			
Regular	20 (19.4%)	127 (18.2%)	0.7650
Irregular	83 (80.6%)	571 (81.8%)	
Margin			
Smooth	35 (34.0%)	233 (33.4%)	0.9040
Ill-defined	68 (66.0%)	465 (66.6%)	
Vascularity			
Avascular or minimal blood flow	44 (42.7%)	237 (34.0%)	0.0819
Moderate or marked intranodular vascularity	59 (57.3%)	461 (66.0%)	
Needling direction			
Transisthmic needle insertion	49 (47.6%)	382 (54.7%)	0.1740
Lateral cervical needle insertion	54 (52.4%)	316 (45.3%)	
Experience of the operator			
Inexperienced	85 (82.5%)	505 (72.3%)	0.0286
Experienced	18 (17.5%)	193 (27.7%)	
Number of needle passes			
3 times	69 (67.0%)	398 (57.0%)	0.0554
4 times	34 (33.0%)	300 (43.0%)	

ND, nondiagnostic.

**Table 4:** Multivariate analysis of affecting factors with logistic regression in ultrasound-guided fine-needle aspiration for thyroid nodules.

Variables	Β	Odds Ratio [95% CI]	*p* Values
Hashimoto’s thyroiditis	0.5934	1.810 [1.076, 3.045]	0.0254
Orientation	0.7121	2.038 [1.260, 3.296]	0.0037
Calcification	0.5692	1.767 [1.115, 2.799]	0.0153
Number of needle passes	0.5595	1.750 [1.094, 2.799]	0.0196
Experience of the operator	0.5711	1.770 [1.005, 3.118]	0.0481

CI, confidence interval.

## Data Availability

The data presented in this study are available on request from the corresponding author. The data are not publicly available due to ethical restrictions.
